# Circadian misalignment alters insulin sensitivity during the light phase and shifts glucose tolerance rhythms in female mice

**DOI:** 10.1371/journal.pone.0225813

**Published:** 2019-12-18

**Authors:** Li-Xin Zhong, Xiao-Na Li, Guang-Yu Yang, Xia Zhang, Wen-Xue Li, Qian-Qian Zhang, Huan-Xin Pan, Hui-Hong Zhang, Meng-Ya Zhou, Yi-Ding Wang, Wei-Wei Zhang, Qian-Sheng Hu, Wei Zhu, Bo Zhang

**Affiliations:** 1 Department of Occupational and Environmental Health, School of Public Health, Sun Yat-sen University, Guangzhou, Guangdong, China; 2 Department of Toxicology, Guangzhou Center for Disease Control and Prevention, Guangzhou, Guangdong, China; 3 Food Safety and Health Research Center, School of Public Health, Southern Medical University, Guangzhou, Guangdong, China; University of Lübeck, GERMANY

## Abstract

Shift work and jet lag, characterized by circadian misalignment, can disrupt several physiological activities, but whether they affect the rhythm of glucose uptake and insulin sensitivity remain unclear. In the present study, female C57BL/6J mice were maintained for four weeks under the condition of 8-hour phase advance and delay every 3–4 days to mimic shift work. Intraperitoneal glucose tolerance test (IPGTT) and intraperitoneal insulin tolerance test (IPITT) were performed repeatedly at Zeitgeber time (ZT) 0, ZT6, ZT12, and ZT18. Glucose-stimulated insulin secretion (GSIS) test was performed at ZT6. We found that the average level of daily glucose tolerance did not decrease but the phase of glucose tolerance advanced by 2.27 hours and the amplitude attenuated by 20.4% in shift work mice. At ZT6, IPITT showed blood glucose at 30 min after insulin injection decreased faster in shift work mice (**−**3.50±0.74mmol/L, **−**61.58±7.89%) than that in control mice (**−**2.11±1.10mmol/L, **−**33.72±17.24%), but IPGTT and GSIS test showed no significant difference between the two groups. Food intake monitor showed that the feeding time of shift work mice continued to advance. Restricting feed to a fixed 12-hour period alleviated the increase of insulin sensitivity induced by shift-work. We also observed that an increase of blood glucose and liver glycogen at ZT0, as well as a phase advance of liver clock genes and some glucose metabolism-related genes such as forkhead box O1 (*Foxo1*) and peroxisome proliferator activated receptor alpha (*Pparα*) in shift work mice. Our results showed that light change-simulated shift work altered insulin sensitivity during the light phase and shifted glucose tolerance rhythms in female mice, suggesting a causal association between long-term shift work and type 2 diabetes.

## Introduction

Biological clocks, functioning in the suprachiasmatic nuclei (SCN) of the hypothalamus and peripheral organs/tissues, such as liver, muscle, adipose and pancreas, play a pivotal role in regulating glucose homeostasis [1]. Light is the most important entrainment factor for SCN to synchronize the central rhythm with environmental light/dark changes and diurnal fluctuation of blood glucose. After receiving information transmitted from the SCN, peripheral organs orchestrate daily oscillations in glucose uptake, insulin release and insulin sensitivity to adapt to the external light-dark environment [2, 3].

Clock genes are essential to maintain glucose-insulin homeostasis. The loss or mutation of clock genes leads to circadian disruption and causes serious metabolic problems [4–7]. For instance, whole-body inactivation of brain and muscle ARNT-like 1 (*Bmal1*) or circadian locomotor output cycles kaput (*Clock*) could suppress glucose rhythm, impair gluconeogenesis, and inhibit glucose recovery in insulin-induced hypoglycemia [4]. Global *Clock* mutant mice were found to readily develop metabolic disorders and obesity [5]. Global *Bmal1*-knockout mice showed a lack of insulin sensitivity rhythm, resistance to insulin and impairment in glucose-stimulated insulin secretion [6, 7]. Several tissue-specific clock gene deletion studies further showed there were direct associations between peripheral clocks and glucose metabolism independent of the central clock [8–11]. Liver *Bmal1*-knockout led to hypoglycemia in the fasting phase and induced an abnormal increase in glucose clearance ability [8]. Muscle *Bmal1*-deletion caused glucose intolerance and hypoglycemia in the non-fasting state, and led to muscle-specific damage in insulin-stimulated glucose uptake [9, 10]. Pancreatic-specific *Bmal1*-knockout mice showed glucose intolerance and abnormal glucose-stimulated insulin secretion [11]. These studies revealed that the circadian clock controls multiple components of glucose metabolism and supported the causal association between circadian disruption caused by shift work or jet lag and type 2 diabetes mellitus (T2DM).

Repeated light-dark (LD) phase shift schedules were usually used to mimic environmental circadian misalignment such as shift work and jet lag [12]. Unlike the aforementioned transgenic mouse models, light/dark phase shifts could continually affect the phase of many physiological parameters such as body temperature, heart rate and movement [13]. However, to our knowledge, it remains unclear whether light-induced circadian disruption could alter the phase of glucose tolerance or insulin sensitivity. Previous studies investigating the effects of LD phase shifts on glucose/insulin tolerance showed inconsistent results. Some studies reported that LD phase shifts induced glucose intolerance [14–16]. However, Bartol et al. found that exposure to shifted LD cycles for five months did not affect glucose tolerance [17]. Gale and his colleagues indicated that repeated LD cycle advances failed to affect glucose-stimulated insulin secretion and insulin sensitivity [18]. It is noteworthy that most of the previous studies measured glucose tolerance and insulin sensitivity at a single time point, which may contribute to the discrepancy because diurnal variations of blood glucose homeostasis were neglected (see S1 Table for more details). Thus, it is necessary to investigate the effect of circadian misalignment on glucose metabolism at multiple time points instead of at one single time point.

To answer the question of whether light-induced circadian misalignment affects the rhythms of glucose metabolism, female C57BL/6J mice were exposed to a recurrent 8-hour phase-shift LD cycle for 4 weeks and glucose homeostasis indicators at multiple time points were analyzed. For convenience, this repeated LD phase shift schedule is simply called shift work in the following description.

## Materials and methods

### Animals

**Fig 1:**
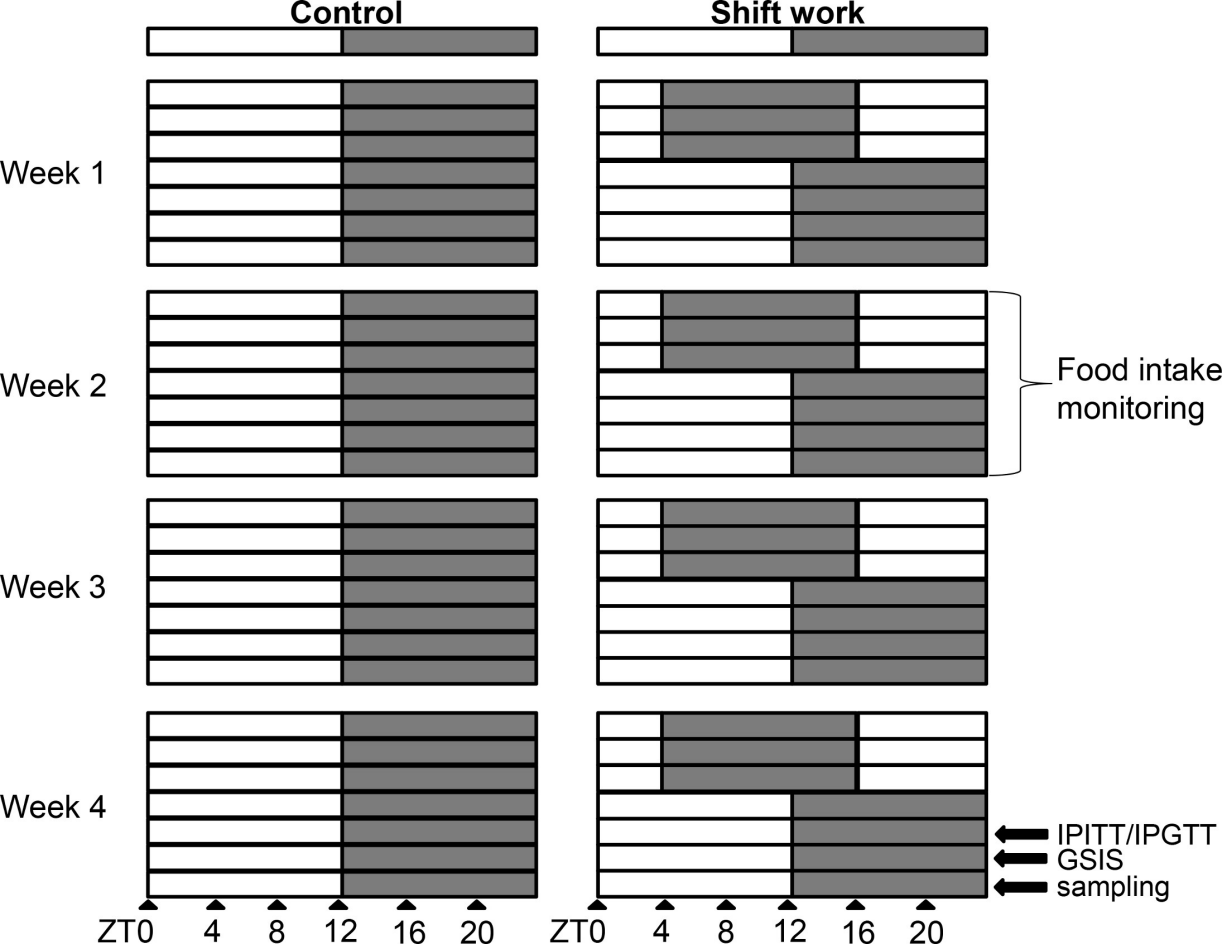
Time schedules of photoperiod protocols over four weeks. In each week, shift work animals were exposed to an 8-h phase advance photoperiod for three days, and then the phase of photoperiod returned to the normal light-dark cycle for four days. Control animals were subjected to a fixed light-dark cycle for four weeks. Light phase is indicated by a white bar while dark phase by a black bar. The timings of blood and tissue sampling are shown as triangles. Notes: ZT: Zeitgeber time, ZT0 represents the time of lights on and ZT12 represents the time of lights off.

Female specific pathogen-free (SPF) C57BL/6J mice were purchased from the Centre of Medical Laboratory Animal of Guangdong, China. All mice were housed in a barrier animal facility (room temperature maintained at 20±2°C and humidity held at 50%–75%) with food and water *ad libitum*. After adaptive feeding in 12-hour:12-hour LD cycles for at least a week, mice aged 6–7 weeks were randomly assigned to the control group or shift work group. The mice in the control group were exposed to a normal LD cycle (lights on at 0700 h and off at 1900 h); and the mice in the shift work group were exposed to repeated light phase shifts for 4–6 weeks. Briefly, in each week, the mice were maintained under an 8-hour light phase advance shift condition for the first 3 days (lights on at 2300 h and off at 1100 h), and then the phase of the light period was recovered to the normal LD cycle for 4 days (Fig 1). Room light was supplied by white cold fluorescent lamps, with 400±100 lx light intensity at the head level. All experimental protocols were reviewed and approved by the Animal Ethics Committee of School of Public Health, Sun Yat-Sen University (No.2017-008). Animals were killed by anesthesia with pentobarbital sodium, and all efforts were made to minimize their suffering.

### Intraperitoneal insulin tolerance test (IPITT) and glucose-stimulated insulin secretion (GSIS) test

IPITT was performed on the fifth day of week 4. We chose this day to let shift work mice be acclimated to a normal LD cycle for 24 hours before the test. Briefly, the mice were fasted for 4 hours and then injected intraperitoneally with insulin (0.75 IU/kg b.w.; Novo Nordisk, Bagsværd, Denmark; n = 7-10/group/time point) at ZT0, ZT6, ZT12 and ZT18 (ZT: Zeitgeber time, ZT0 represents the time of lights on, and ZT12 represents the time of lights off). The tail top blood was collected to measure glucose (Accu-Chek Performa, Roche Holding AG, Basel, Switzerland) prior to injection and at the specific time points after injection.

To assay the impact of shift work on the function of pancreatic β cells, the GSIS test was performed at ZT6 on the sixth day of week 4. After 13 hours of fasting, the mice (n = 10/group) were injected with glucose (2 g/kg b.w.) intraperitoneally, tail blood was collected at 0, 15 and 30 minutes, and plasma was immediately separated and stored at **−**80°C. Insulin levels were measured using Rat/Mouse Insulin ELISA Kit (MilliporeSigma, Burlington, MA, USA).

### Locomotor activity assay

On the fifth day of week 5, a separate cohort of mice (n = 4/group) was placed in white square chambers (length*width = 37 cm*37 cm) surrounded by 50-cm-high white plastic sheets. Video-tracking cameras (open field test analysis system; Flyde, China) were placed above the chamber, which continuously monitored mice’s movements throughout the whole day. Total moving distance over 24 hours (meter per hour) was calculated for each mouse. Food and water were freely available during the test. All mice were acclimated to the chambers for 12 hours before tracking.

### Food intake monitor

In total, 20 mice (n = 10 mice/group) were singly housed in a metabolic cage in week 2. The chow weight was recorded every 6 hours from ZT0 of the first day to ZT24 of the seventh day.

### Intraperitoneal glucose tolerance test (IPGTT)

To compare the circadian rhythm of glucose uptake between the control and the shift work groups, glucose tolerance tests were performed at ZT0, ZT6, ZT12 and ZT18 on the fifth day of week 4. Briefly, the mice were fasted for 16 hours before injected intraperitoneally with D-(+)-glucose (2g/kg, Sigma-Aldrich, St. Louis, MO, USA; n = 7–10/group/time point). Blood glucose was measured repeatedly at 0, 15, 30, 60, 90, and 120 min after glucose injection.

### Blood and tissue collection

After the IPGTT experiment, the mice were recovered for two days. Biological specimens were collected on the last day of week 4. The mice (n = 5/group/time point) were administered with 1% pentobarbital sodium intraperitoneally and sacrificed at ZT0, ZT4, ZT8, ZT12, ZT16, or ZT20. All mice were fasted for 12 hours before sacrifice in order to reduce the effects of food intake. Abdominal venous blood was collected and centrifuged for 10 minutes at 3000 rpm. The plasma was stored at **−**80°C until analyses. The liver was harvested, weighed, and frozen in liquid nitrogen for a few minutes and then was stored at **−**80°C until further use. The gonadal fat and pancreas were quickly separated and weighed. During the dark phase, the experiments were performed in a weak red-light environment (<5 lx).

### Time-restricted feeding (TRF) treatment

After 4 weeks, a subset of mice (n = 7 in the control group and n = 11 in the shift work group) were continuously exposed to the designed photoperiod for additional 11 days. During this extended period, food was only available during 12 hours per day. For comparison between the two groups the feeding time was set as 19:00–7:00, i.e. ZT12–24 in normal LD cycles (S1 Fig). On day 12, the IPITT was performed at ZT6 as mentioned above.

### Biochemical analysis

Plasma glucose was measured with an automatic biochemical analyser (Drew Scientific Inc, Miami Lakes, FL, USA). Plasma insulin was measured using the Rat/Mouse Insulin ELISA Kit (MilliporeSigma, Burlington, MA, USA). Plasma glucagon was measured using the Glucagon ELISA-10μL kit (Mercodia AB, Uppsala, Sweden). The concentrations of corticosterone were measured with a Corticosterone Competitive ELISA Kit (ThermoFisher, San Diego, CA, USA). The hepatic glycogen content was measured using a commercial glycogen assay kit (Nanjing Jiancheng Bioengineering Institute, Nanjing, China).

### Homeostasis model assessment of insulin resistance (HOMA-IR) index

The HOMA-IR indices were calculated to evaluate insulin sensitivity. Fasting plasma glucose and insulin concentrations were obtained from the blood samples analysis at ZT0 to ZT20 as mentioned above. The calculation formula is as following: HOMA-IR = (fasting plasma insulin (mIU/L)) * (fasting plasma glucose(mmol/L))/22.5 [19]

### Gene expression analysis of liver

Hepatic total RNA was isolated with TRIzol reagent (Life Technologies, USA). First-strand cDNA was reversely transcribed from approximately 500 ng of RNA using the PrimeScript^TM^ RT Master Mix (Takara Bio Inc., Kusatsu, Shiga Prefecture, Japan) according to the manufacturer’s instructions. Real-time PCR was performed using the SYBR Premix Ex Taq^TM^II kit (Takara Bio Inc., Kustsu, Shiga Prefecture, Japan). The gene expression levels were measured using the Applied Biosystems Quant Studio^TM^ 7 Real Time PCR machine (Thermo Fisher, San Diego, CA, USA) in double copies. β-actin was used as the internal control. The relative expression of genes was calculated with the 2 ^-ΔΔCt^ method, and the daily expression profiles were presented as the fold change compared to the mean level of the control group at ZT0. The primer sequences were listed in S2 Table.

### Statistical analysis

All data were presented as the means ± standard deviation (SD). The glucose levels in the IPITT/IPGTT were analyzed with two-factor (time*treatment) repeated-measures analysis of variance (ANOVA) and Bonferroni's post-hoc tests were used for pairwise comparisons. The comparisons of the circadian data with respect to the area under the curve (AUC) of IPGTT and area above the curve (AAC) of IPITT, HOMA-IR, metabolites, hormones and gene expression levels between two groups were performed with two-factor (time*treatment) ANOVA and Bonferroni's post-hoc tests. Student’s *t* test was also applied if necessary. AUC and AAC were calculated with GraphPad Prism 5.0 (GraphPad Software, Inc., San Diego, CA, USA). The baselines of glucose varied during the day; therefore; all glucose values of tolerance test were normalized by deducting the baseline value in t = 0 minutes for better comparison. SPSS20.0 was used, and *P*<0.05 was considered a significant difference. The figures were plotted with GraphPad Prism 5.0.

Cosinor analysis was performed to test whether diurnal rhythmicity existed (SigmaPlot 14.0; Systat Software Inc., San Jose, CA). Data were fitted to a cosine equation as follows:

$$y=A+Bcos\left[\frac{2\pi }{24}(x-C)\right]$$ (1)

Here, A is the mesor (midline estimating statistic of rhythm), B is the amplitude, and C is the phase of maximum (unit is hours).

## Results

### Shift work disturbs plasma glucose levels and hepatic glycogen content

**Fig 2:**
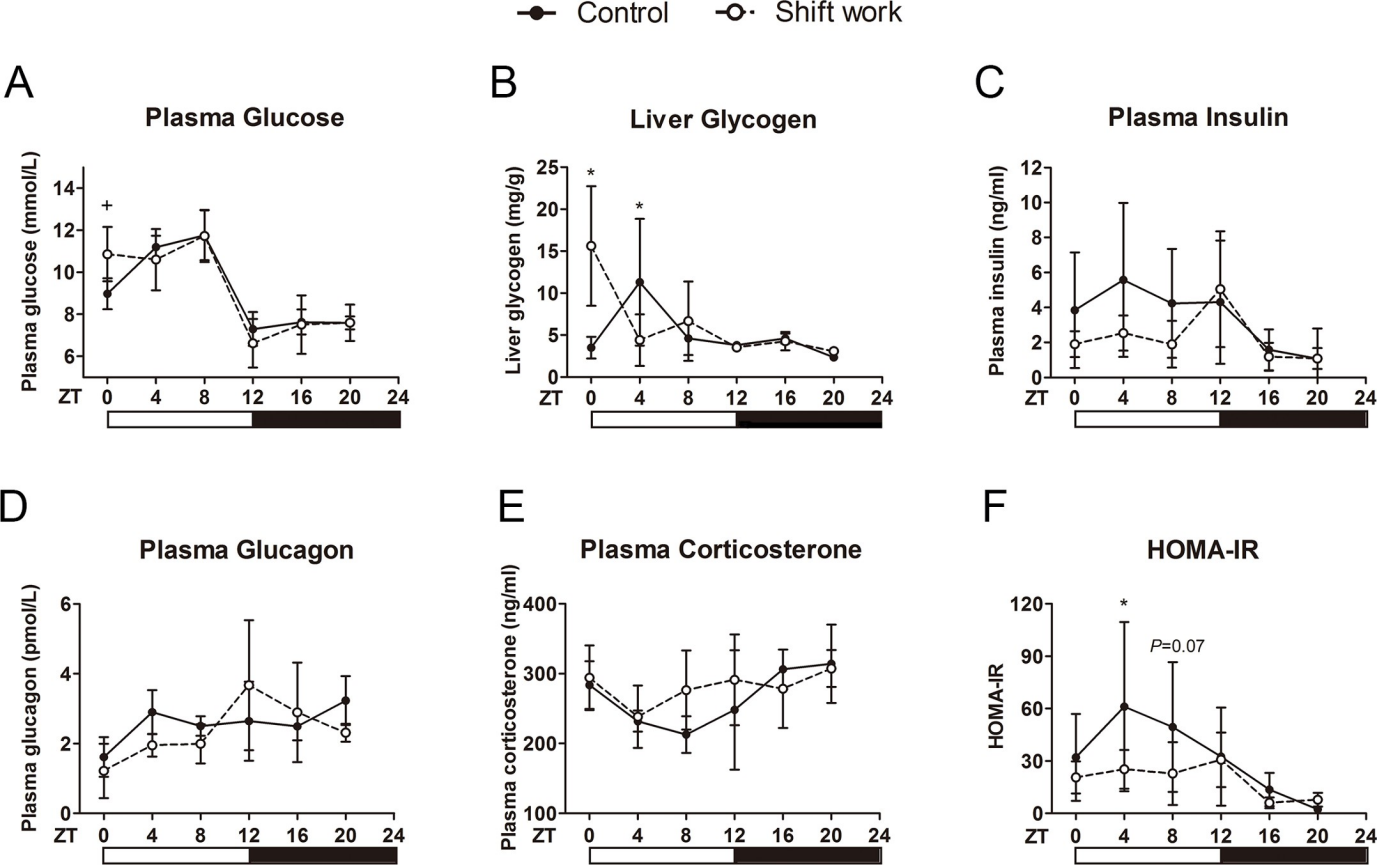
The effect of shift work on blood biochemistry, liver glycogen and HOMA-IR. (A) Plasma glucose, and (B) liver glycogen and plasma (C) insulin, (D) glucagon, (E) corticosterone, (F) homeostasis model assessment of insulin resistance index (HOMA-IR) were detected across a 24-h cycle in control and shift work mice. N = 4–5/time point/group, except glucose and HOMA-IR in the control group at ZT20 (n = 3). The data are mean ± SD. *P<0.05, Two-way ANOVA and Bonferroni post hoc test; +*P*<0.05, Student’s *t* test.

The shift work mice were subjected to repeated 8-hour light phase advance and delay every 3–4 days for four weeks. Shift work did not affect body weight, and organ coefficients of liver, gonadal white adipose tissue (WAT), and pancreas (S3 Table). As shown in Fig 2A, shift work mice displayed similar diurnal fluctuation as control mice in the level of fasting plasma glucose (*P*>0.05, two-way ANOVA). However, compared with the control, the glucose level of shift work mice showed a significant increase at ZT0 (10.86±1.29 mmol/L in shift work mice vs 8.98±0.74 mmol/L in control mice; *t* = 2.821, *P* = 0.022; Fig 2A). We next investigated whether the levels of hepatic glycogen (the main source of fasting blood glucose) and gluco-regulatory hormones were affected by shift work. The results showed that the timing profiles of plasma insulin, glucagon and corticosterone also did not be affected by shift work (Fig 2C, 2D and 2E, respectively). Shift work treatment dramatically increased the hepatic glycogen content at ZT0 (*F* = 29.883, *P*<0.001; Fig 2B), while the levels of hepatic glycogen in shift work mice were significantly lower than those in control mice at ZT4 (*F* = 9.744, *P* = 0.003).

### Shift work impacts the temporal expression of clock genes and glycometabolism-related genes in the liver

**Fig 3:**
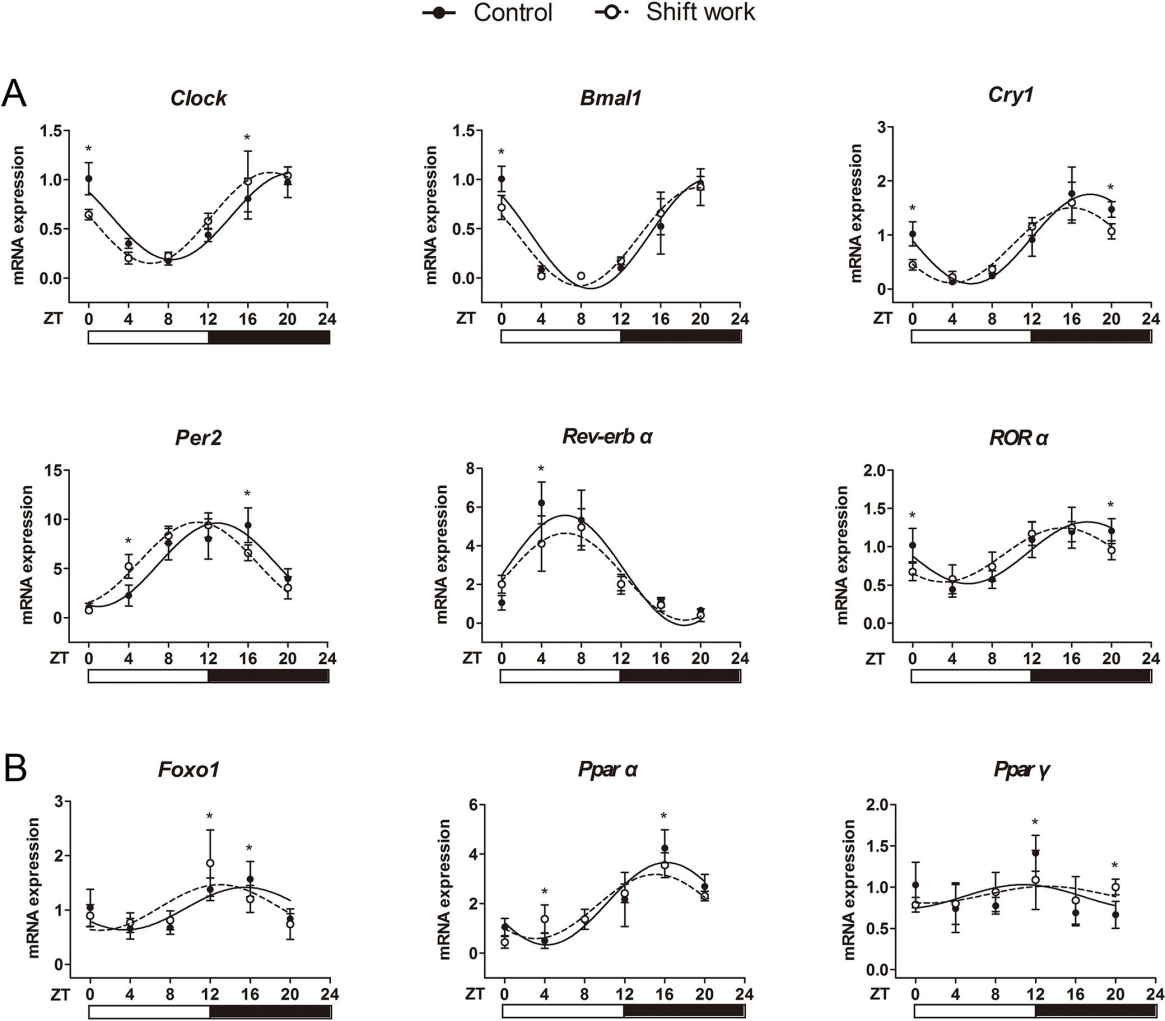
The effect of shift work on clock genes and glycometabolism-related genes in the liver. Transcript profiles of (A) clock genes and (B) glycometabolism-related genes of the liver were analyzed by qPCR. N = 5/time point/group, except *Pparα* in the shift work group at ZT4 (n = 4). The data are mean ± SD. Two-way ANOVA with Bonferroni post hoc test were performed. *P<0.05 showed significant difference between two groups at the corresponding time point.

The liver plays a pivotal role in maintaining systemic glucose homeostasis. To further investigate the effects of shift work on glucose metabolism, we examined the expression profile of circadian genes and several key genes involved in gluconeogenesis (glucose 6-phosphatase (*G6pase*), phosphoenolpyruvate carboxykinase (*PEPCK*)), glucose transport (glucose transport 2 (*Glut2*)), glycogenolysis (liver glycogen phosphorylase (*Pygl*)), glycogen synthesis (glycogen synthase 2 (*Gys2*)), glycolysis (glucokinase (*Gck*)) and the corresponding transcription factors (forkhead box O1 (*Foxo1*), peroxisome proliferator activated receptor alpha (*Pparα*), *Pparγ* and PPAR gamma coactivator (*PGC-1α*)) in the mouse liver. Quantitative PCR results showed that most genes displayed significant oscillations (*P*<0.05) in both normal and shift work mice. Among them, the rhythms of circadian gene expressions in the shift work group were significantly different from those in the control group (Fig 3A). Cosinor analysis showed a phase advance and amplitude attenuation of clock genes in shift work mice (phase: *Clock*:**−**2.07 h, *Bmal1*:**−**1.15 h, *Cry1*:**−**1.85 h, *Per2*:**−**1.99 h, and *RORα*:**−**2.48 h; amplitude: *Bmal1*:**−**10.7%, *Cry1*:**−**15.7%, *Per2*:**−**1.9%, *Rev-erbα*:**−**20.8%, *RORα*:**−**12.5%). Moreover, the daily variations in the expression levels of the *Foxo1*, *Pparα* and *Pparγ* genes also exhibited a significant difference between the two group (*Foxo1*: *F*_interaction_ = 2.706, *P* = 0.031; *Pparα*: *F*_interaction_ = 3.207, *P* = 0.014; *Pparγ*: *F*_interaction_ = 3.164, *P* = 0.015; two-way ANOVA; Fig 3B). A phase advance of *Foxo1* and *Pparα* were also observed (**−**2.54 h and **−**1.06 h, respectively), but only *Pparα* showed an amplitude attenuation (**−**21.7%). As to the *Pparγ* expression, no circadian rhythms were observed in both the shift work and control groups (shift work: *t* = 1.611, *P*>0.05; control: *t* = 1.734, *P*>0.05). There was no significant difference in the 24-hour expression profiles of other genes related to glucose metabolism (*G6pase*, *PEPCK*, *Glut2*, *Pygl*, *Gys2*, *Gck* and *PGC-1α*) between the two groups (S6 Table).

### Shift work increases the insulin sensitivity of the light phase

**Fig 4:**
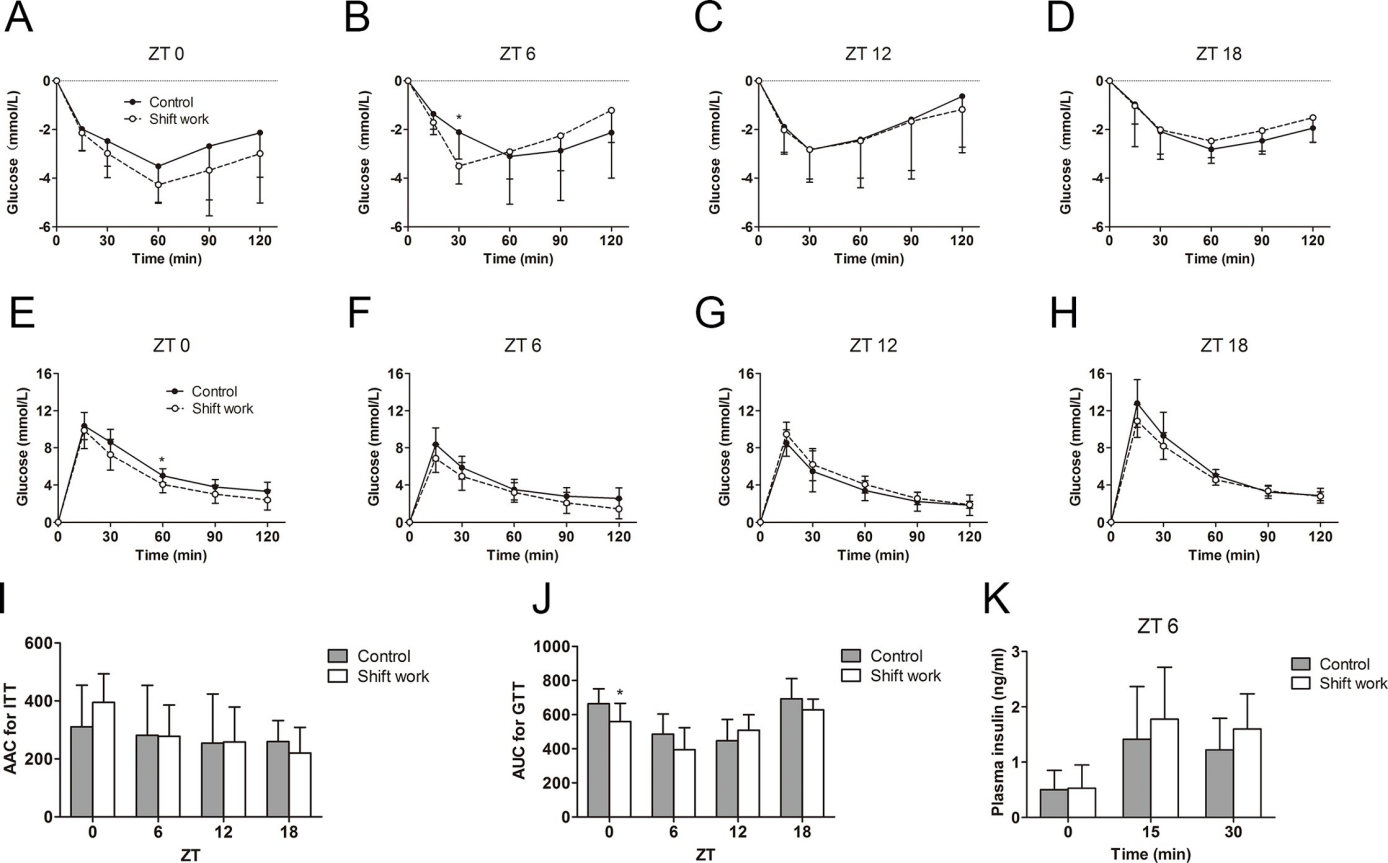
The effects of shift work on insulin sensitivity and glucose tolerance during the day. (A–D) Insulin tolerance tests (ITT) and (E–H) glucose tolerance tests (GTT) in control mice and shift work mice at different ZT were performed. (I) Area above the glucose curve for ITT and (J) area under the glucose curve for GTT was calculated for each mouse. (K) Glucose-stimulated insulin secretion test was performed for control and shift work mice at ZT6. N = 7–10/time point/group. The data are shown as mean and SD. Statistically significant differences were detected by two-way repeated measures ANOVA with Bonferroni's post hoc test (A–H) and by two-way ANOVA coupled with Bonferroni's post hoc test (I–K). *P<0.05.

The effect of shift work on HOMA-IR was displayed in Fig 2F. Two-way ANOVA results indicated a significant treatment effect (*F* = 4.356, *P* = 0.043). The post-hoc test displayed a significantly lower HOMA-IR index at ZT4 (*F* = 5.046, *P* = 0.030) and a borderline significant decrease at ZT8 (*F* = 3.468, *P* = 0.070) in the shift work group as compared with those of the control group. No significant differences were observed at other ZTs. These results suggested that shift work could increase peripheral insulin sensitivity in the middle of light period. To verify this hypothesis, the IPITT at ZT0, ZT6, ZT12 and ZT18 were performed. Consistent with the HOMA-IR results, two-way repeated measures ANOVA found a significant effect of interaction at ZT6 (*F* = 6.136, *P* = 0.007; Fig 4B). The post-hoc test also showed that the glucose level at 30 minutes post-injection was significantly lower in shift work mice (**−**3.50±0.74mmol/L, **−**61.58±7.89%) than that in control mice (**−**2.11±1.10mmol/L, **−**33.72±17.24%) at ZT6 (*F* = 9.056, *P* = 0.009), suggesting that shift work mice are more sensitive to insulin at ZT6. Glucose levels at other time points in the IPITT curve did not display a significant difference between the two groups. The temporal variation data of AAC of IPITT were not analyzed because the time of the minimum value of the insulin tolerance curve was different among the ZTs, which meant the AAC of the curve did not adequately reflect the real fluctuations in insulin sensitivity (Fig 4A–4D and 4I).

### Shift work advances the phase of the glucose tolerance rhythm

IPGTT were performed at ZT0, ZT6, ZT12 and ZT18 on the same day as IPITT. The AUC of the IPGTT of both groups displayed a temporal variation (both *P*<0.001). In control mice, the minimum AUC value appeared at the start of the dark phase (ZT12) and was significantly lower than that of ZT0 and ZT18 (*P* = 0.001 and *P*<0.001, respectively; Fig 4J). However, in shift work mice, the minimum AUC value occurred at ZT6. Statistical analysis showed that the AUC of ZT6 was significantly lower than that of ZT0 and ZT18 (*P* = 0.006 and *P*<0.001, respectively), while no significant difference was observed in the AUC value at ZT12 as compared to that at ZT0, ZT6 or ZT18 in shift work mice. This result suggested that shift work caused a peak advance in glucose tolerance rhythm. The cosinor analysis showed that the AUC of both the control and shift work groups displayed significant rhythmicity (control: *t* = 5.843, *P*<0.001; shift work: *t* = 5.243, *P*<0.001). The AUC of the control group peaked late (at ZT 21.09), while shift work mice displayed a phase advance of the AUC rhythm by 2.27 hours (peak at ZT 18.82). The amplitude was attenuated by 20.4% in shift work mice. The mesor of AUC was 8.6% lower in shift work mice, reflecting a slightly strengthened glucose tolerance.

Furthermore, glucose tolerance was also compared at each time point. Two-way ANOVA showed a significant treatment effect (*F* = 4.230, *P* = 0.043). The post hoc analysis on AUC further revealed that shift work mice had a higher glucose tolerance at ZT0 than control mice (*F* = 4.757, *P* = 0.033; Fig 4J). After 60 minutes of intraperitoneal injection of glucose at ZT0, the blood glucose clearance rate in shift work mice showed a statistically significant improvement (*F* = 6.761, *P* = 0.018; Fig 4E). Unexpectedly, the AUC of the IPGTT at ZT6 did not differ between the shift work and control mice (*F* = 3.451, *P* = 0.067; Fig 4F), suggesting that the increase in insulin sensitivity induced by shift work is insufficient to improve the glucose tolerance in mice.

At ZT6, we performed GSIS to evaluate insulin response function of islet β cells. The quantity of insulin release in response to glucose injection displayed a modest but statistically insignificant augmentation in shift work mice in comparison with that in control mice (*F* = 0.383, *P*>0.05; Fig 4K). Taken together, the above results revealed that an enhancement in insulin sensitivity at ZT6 in shift work mice was not coupled with an increase in islet function and glucose tolerance.

### Shift work advances the phase of food intake rhythm

**Fig 5:**
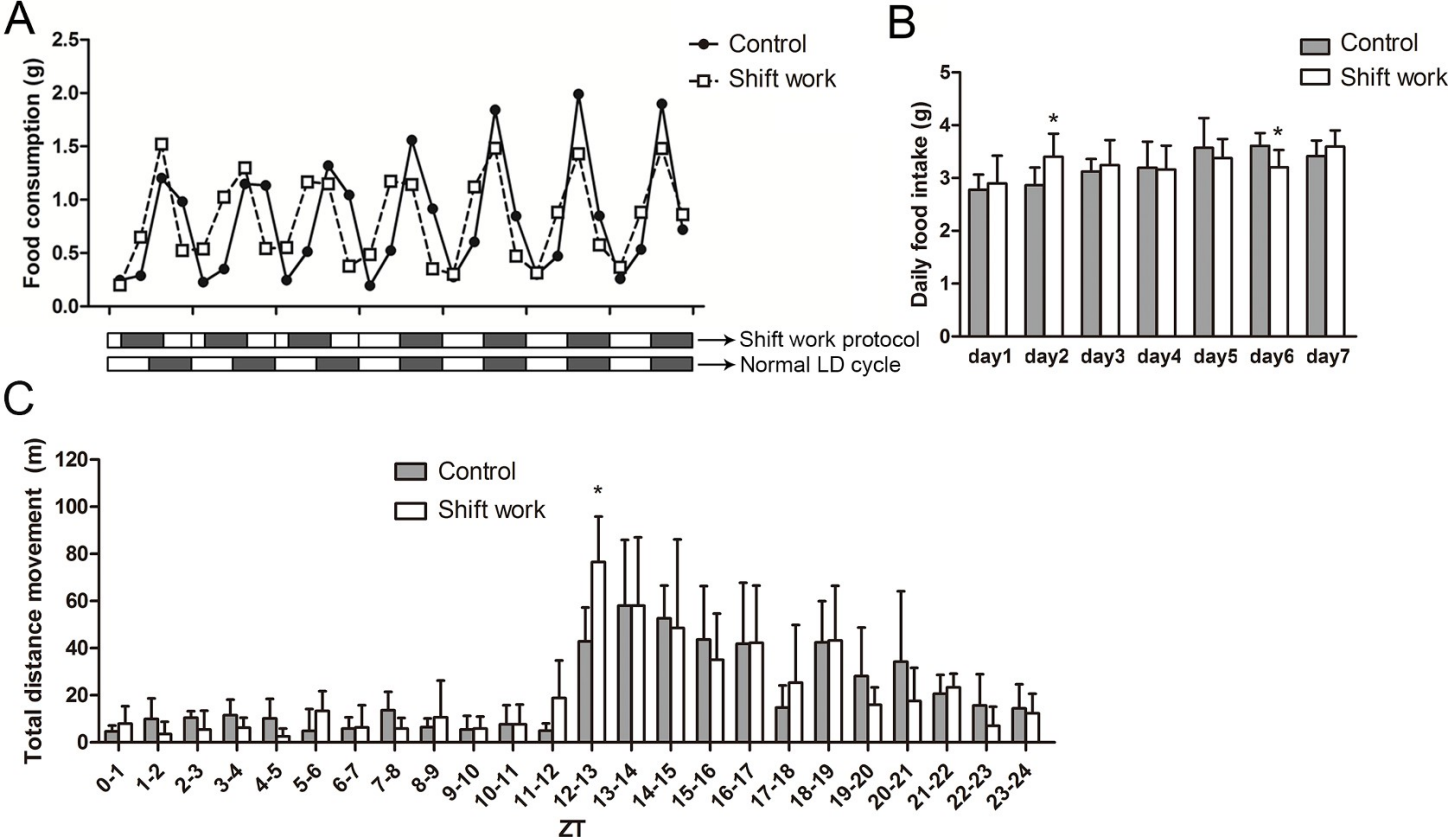
The effect of shift work on feeding rhythm and activity rhythm. (A) Food intake rhythms in control and shiftwork mice were monitored over a seven-day period. One scale on the X-axis represents one day. Corresponding light-dark patterns were exhibited as bars below the X-axis (up: shift work condition; down: normal light-dark (LD) condition), in which the light phase is indicated by a white bar while the dark phase is indicated with a black bar. The data are mean values from 8–10 mice in each group. For each day, food intake during ZT0–6, 6–12, 12–18 and 18–24 were represented by 4 consecutive data points, respectively. (B) Total food intake of control and shift work mice over a seven-day cycle. N = 8–10/day/group. (C) Average locomotor activity (meter per hour) in control and shift work mice on the fifth day of the fifth week (n = 4 per group). ZT0–12 represents the light phase and ZT12–24 represents the dark phase. *P<0.05.

There was no significant difference in average daily food intakes between the two groups during the 7-day food intake monitoring period, although there were significant differences on two days (Fig 5B). The chow weight was recorded every 6 hours per day, so that the feeding rhythm could be analysed. As shown in Fig 5A, the shift work mice consistently consumed food earlier than the control mice. Results from cosinor analysis also indicated a phase-advanced effect on food intake rhythms of shift work mice (S4 Table).

### Shift work does not alter the rhythm of voluntary activity

To investigate whether the changes in rhythms of glucose tolerance and insulin tolerance in shift work mice were caused by physical activity, the locomotor activity rhythm was monitored for a 24-hour period. As shown in Fig 5C, all mice's activity increased at ZT12. There was no significant difference in 24-hour total movement distance, light-phase movement distance and dark-phase movement distance between the two groups (S5 Table). The phase of the activity rhythm in shift work mice did not advance as the phase of glucose tolerance advanced by 2.27 hours and the insulin sensitivity increased at ZT6. These results suggested that the change in glucose tolerance and insulin sensitivity in shift work mice might not be caused by physical activity.

### Time-restricted feeding withdraws the enhancement of insulin sensitivity of the light phase induced by shift work

**Fig 6:**
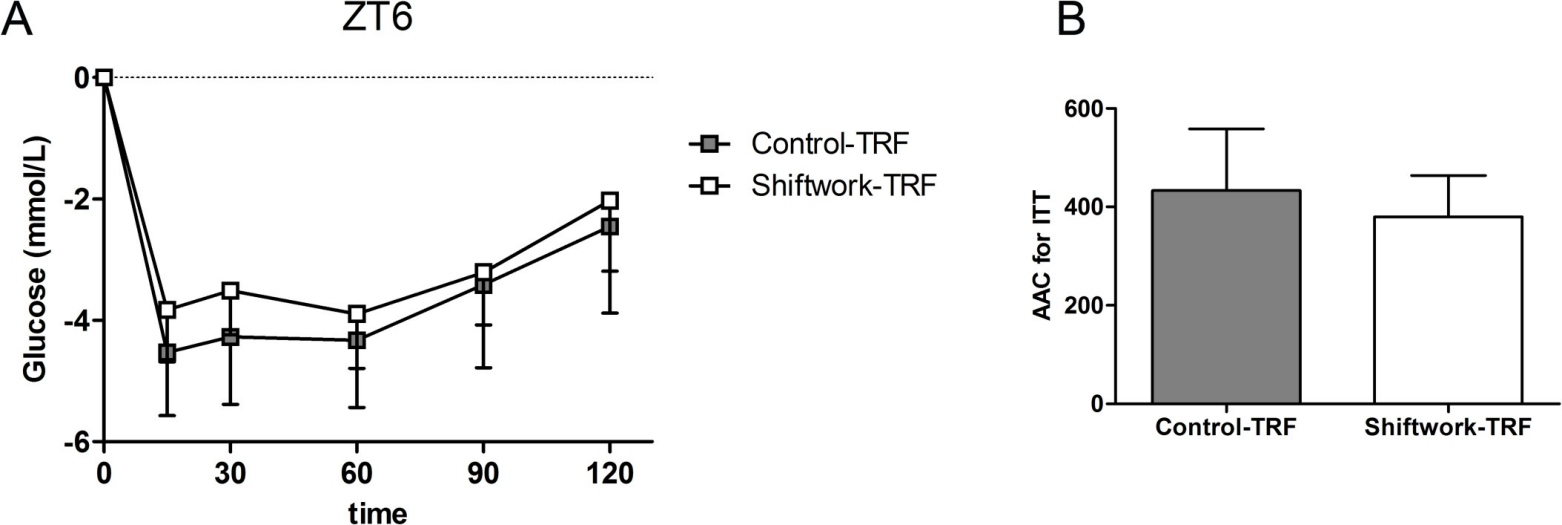
Time restricted feeding withdrew shift work effects on insulin sensitivity of the light phase. (A) Insulin tolerance tests (ITT) of Control-TRF (n = 7) and Shiftwork-TRF (n = 11) mice at ZT6 were performed. Two-way repeated measures ANOVA (post-injection time*treatment) were performed. No significant difference between groups was observed. (B) Area above curve (AAC) of ITT was calculated for each mouse. The data are mean±SD. Student’s *t* test was used to detect statistical significance. The AAC of shift work mice are not different from that of control mice under time-restricted feeding condition. TRF: time-restricted feeding.

Given that the increased insulin sensitivity at ZT6 may be an adaptive response to the increased food intake during the light phase, we performed time-restricted feeding intervention to test the hypothesis. During the extended 11 days after the 4-week experiment food was only available during 12 hours per day. The IPITT tests at ZT6 on 12^th^ day showed no significant difference between the two groups (Fig 6). These results indicated that the enhanced insulin sensitivity at ZT6 in shift work mice might be attributed, at least partly, to the phase advance of food intake.

## Discussion

The present results showed that, 4-week shift work increased the levels of fasting blood glucose and hepatic glycogen in the early stages of the light period. Furthermore, peripheral insulin sensitivity was enhanced in the middle of the light period. The augmentation of insulin sensitivity could be reversed by time-restricted feeding. Shift work led to a phase advance and an amplitude attenuation of glucose tolerance rhythms. Similarly, the daily pattern of food intake and expression profiles of liver clock genes and glucose metabolism-related genes (*Foxo1* and *Pparα*) reached the peak value earlier in the shift work mice than in the control mice.

During short-term fasting, circulating glucose is mainly supplied by glycogenolysis and is regulated by gluco-regulating hormones, such as insulin, glucagon, and corticosterone. We detected the 24-hour fasting blood glucose level and found that the blood glucose level of shift work mice increased at ZT0. There was no difference in the levels of gluco-regulating hormones at ZT0, but the content of liver glycogen in the shift work mice was higher at that time, indicating that the increase of fasting blood glucose in the shift work mice may be associated with the increase of liver glycogen content. However, expression of *Gys2* and *Pygl* mRNA in the liver remained unchanged, suggesting that metabolic pathways of liver glycogen mobilization were neither activated nor inhibited by shift work. The most likely explanation for increased blood glucose and hepatic glycogen is the phase advance of feeding time, which made the shift work mice intake more food before the overnight fasting period.

Phase shifts of core clock genes in the liver indicated that shiftwork-like lighting pattern induced a change of circadian rhythm in the liver. Surprisingly, a majority of metabolic genes selected did not show a significant change in our study. It is not consistent with the results from Barclay et al. which showed a global disruption of gene rhythms involved in carbohydrate metabolism in shift work C57BL/6J mice [20]. This discrepancy may be due to the differences in study design, such as the gender of the animal and variation in experimental models used to induce circadian misalignment. Time sleep restricted treatment, performed by Barclay et al., may be a more effective experimental scheme to disturb glucose metabolism.

Time restricted feeding is widely known to be beneficial for the maintenance of glucose homeostasis, mainly due to its ability to reverse the adverse metabolic outcomes of high-fat diet, such as obesity and glucose intolerance [21, 22]. For mice fed with normal diet, limiting food intake to the dark phase did not show significant effects on the amount of food intake, body weight, and glucose metabolism [21, 22]. In a long photoperiod model, Shamsi et al. reported that mice fed with normal chow during the light period for 4 weeks showed increased glucose tolerance and insulin sensitivity [23], which is similar to the result of this study, that is, an increase in feeding during the light phase contributed to an increase in insulin sensitivity. We speculate that this unexpected change in glucose metabolism is an early adaptive response to the variation of food intake time. Light period is the inactive phase of mice and long-term eating in the inactive phase was reported to accelerate weight gain and finally leading to a host of metabolic problems [24, 25]. Nevertheless, in the short term, the body might be able to resist the nutritional challenges by shifting the glucose tolerance rhythm towards unusual feeding periods and increasing insulin sensitivity. Our observations further indicated that keeping feeding time constant for a short term may be sufficient to counteract the abnormal alteration of insulin sensitivity due to the LD shift schedule. Moreover, previous studies demonstrated that restricting food access to the night prevented the disturbance of metabolic rhythms, glucose intolerance and overweight in shift-work nocturnal animals [26–29]. Irregular feeding time seems to be the key mechanistic mediator in the metabolic consequences of photoperiod change. Adjusting meal timing may be an effective way to improve health in shift workers or jet-lagged travelers.

Many epidemiological studies have proven that long-term shift work increases the incidence of T2DM [30–33]. Circadian disorders, insufficient and poor-quality sleep, a lack of physical activity and an unhealthy diet are all identified as the cause of T2DM induced by shift work [34]. Indeed, previous laboratory evidence, both in animals and in humans, has suggested that circadian misalignment itself could induce lower glucose tolerance [14–16, 35, 36]. However, these studies derive their conclusions on test results from one or two time points. We demonstrated, -by performing IPGTT at four time points, -that a 4-week circadian misalignment weakened the rhythmic peak of glucose tolerance, but it did not lead to a decrease in average glucose tolerance. This finding indicated that, at least in the early stages of circadian rhythm disruption, glucose tolerance might not be substantially lowered but merely be a rhythm disorder. From the current data a novel hypothesis can be made, that is, a mismatch between glucose uptake rhythm and meal timings, would affect the postprandial glycemic load in shift workers and fliers crossing time zones. Frequent glucose intolerance during meals likely contributed to the increased risk of T2DM among shift workers.

There are some limitations in our study that should be pointed out. Firstly, we only examined the effects of shift work in female mice. Previous study has shown that female shift workers have a relative lower risk of diabetes than male [30], perturbations caused by shift work in glycometabolism may be more serious in males than in females. The underlying mechanism of gender difference is complicated and remains unclear, but it could be, at least partly attributed to estrogen, which presents a protective effect in shift-work-induced metabolic dysfunctions [37]. Given that the effects of shift work are likely to vary between females and males, a complete, both-sexes design should be done in future studies. Secondly, although the insulin tolerance test exhibited increased systematic insulin sensitivity during the light phase, we cannot identify which insulin-targeted tissue (such as liver, adipose tissue or muscle) plays the most important role in this process. The detection of insulin signaling pathways in all tissues or a hyperinsulinemic-euglycemic clamp experiment needs to be performed in the follow-up study. Thirdly, we did not collect any sleep data. Sleep disorders are also a potential contributor to the adverse cardiometabolic outcomes, sleep-week rhythms observations may be helpful to gain a deeper insight into the underlying mechanisms by which shift work affects glucose metabolism. Finally, clock genes of the SCN should be focused in future studies to examine whether or not the mismatch of SCN and liver clock genes play a role in the impact of circadian misalignment on glucose metabolism and whether time-restricted feeding could improve this mismatch.

In sum, this study provides new insights into the association between shift work and glucose metabolism. We demonstrated for the first time that shift work could increase insulin sensitivity during the light phase and shift the rhythm of glucose tolerance in female mice, and appropriately adjusting meal times might be an effective method for shift workers to alleviate metabolic disorders.

## Supporting information

S1 Fig The schedules of time-restricted feeding regimens. TRF: time-restricted feeding, mice were allowed access to food only in this period (1900h to 0700h). Light phase is indicated by a white bar while dark phase by a black bar. The timing of intraperitoneal insulin tolerance test (IPITT) is shown as triangles. ZT: Zeitgeber time, ZT0 represents the time of lights on and ZT12 represents the time of lights off.
(TIF)

S1 Table Summary of animal studies in which glucose tolerance or insulin sensitivity were detected to investigate the effects of shift work. ZT: Zeitgeber time, ZT0 = lights on, and ZT12 = lights off; OGTT: oral glucose tolerance test; ↓:decreased; NR: not reported.
(PDF)

S2 Table Gene primers for real-time quantitative PCR. (PDF)

S3 Table Body weight and organ coefficients of animals. All values are shown as mean±SD, n = 30 for each group.
(PDF)

S4 Table Cosinor analysis of food consumption rhythms during a full seven-day cycle. Values are mean±SD. Mesor(A): midline estimating statistic of rhythm; Amplitude(B): half of the curve variation range; Phase(C): the timing of curve maximum (unit is hours).
(PDF)

S5 Table Locomotor activity data of animals on the fifth day of cycle. All values are shown as mean±SD, n = 4 for each group. Light phase represented the period of ZT0–12; Dark phase represented the period of ZT12–24.
(PDF)

S6 Table Statistical results of gene expression. The comparisons of gene expression levels between two groups were performed with two-factor analysis of variance (time*treatment) and Bonferroni's post-hoc tests. *P<0.05.
(PDF)
