# Supplementary figures and images for: Circadian misalignment alters insulin sensitivity during the light phase and shifts glucose tolerance rhythms in female mice
Source: PLoS One. 2019 Dec 18;14(12):e0225813. doi: 10.1371/journal.pone.0225813 (PMC6919582; doi:10.1371/journal.pone.0225813)

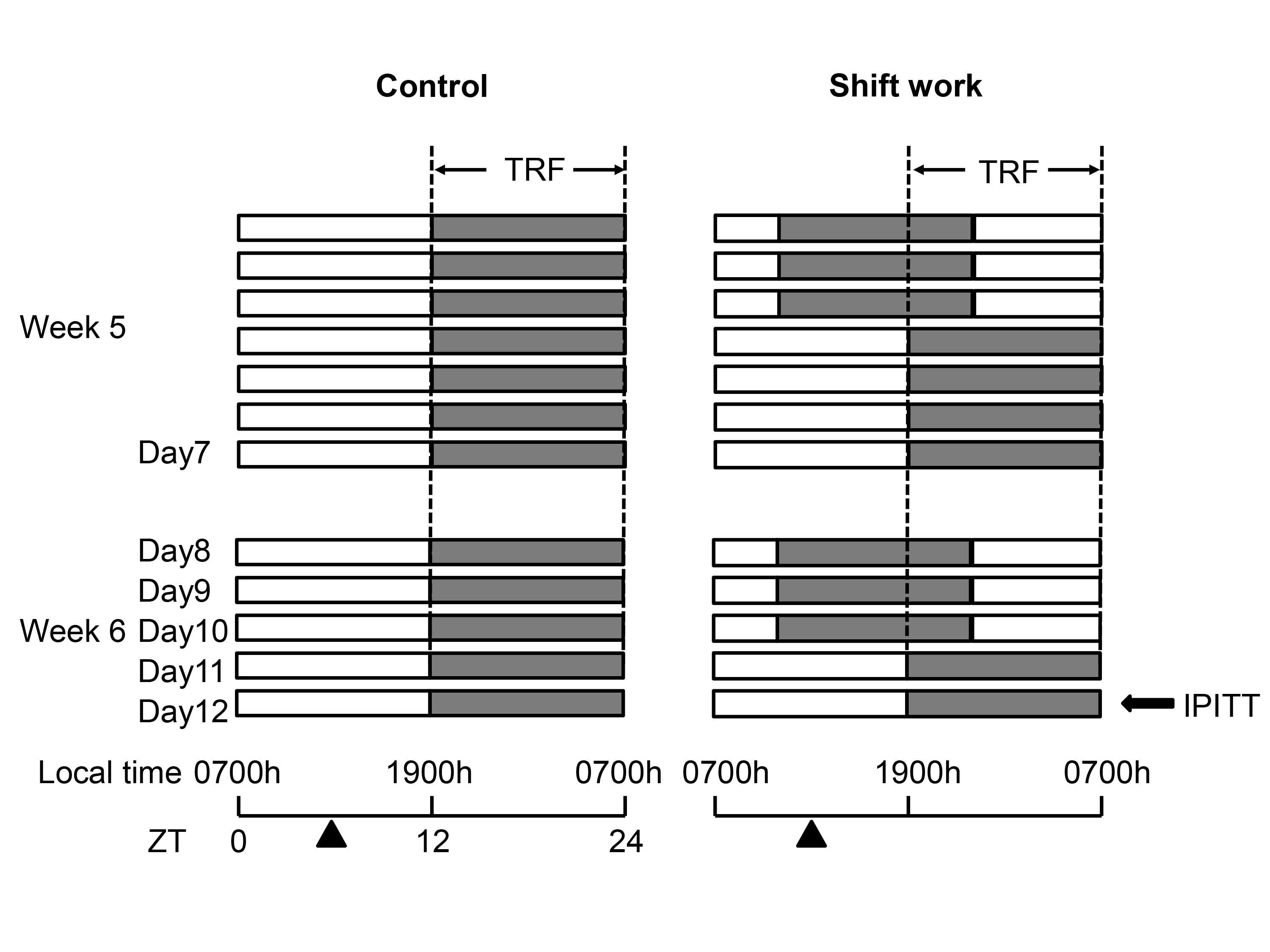

Supplement: S1 Fig — TRF: time-restricted feeding, mice were allowed access to food only in this period (1900h to 0700h). Light phase is indicated by a white bar while dark phase by a black bar. The timing of intraperitoneal insulin tolerance test (IPITT) is shown as triangles. ZT: Zeitgeber time, ZT0 represents the time of lights on and ZT12 represents the time of lights off. (TIF) [file pone.0225813.s001.tif]
