# Supplementary material for: Circadian misalignment alters insulin sensitivity during the light phase and shifts glucose tolerance rhythms in female mice
Source: PLoS One. 2019 Dec 18;14(12):e0225813. doi: 10.1371/journal.pone.0225813 (PMC6919582; doi:10.1371/journal.pone.0225813)
Supplement: S1 Table — ZT: Zeitgeber time, ZT0 = lights on, and ZT12 = lights off; OGTT: oral glucose tolerance test; ↓:decreased; NR: not reported. (PDF) [file pone.0225813.s002.pdf]

7-day shift work cycles	Experimental period	Animal	Glucose tolerance/insulin sensitivity			Study
			Test mothed	Testing time	Outcome	
12 h advance on day 1, normal phase on day 2-7	3 weeks	C57BL/6 male mice	OGTT	ZT9 on day 2	glucose tolerance ↓	Figueiro et al. 2017 [14]
12 h advance on day 1-3, normal phase on day 4-7	3 weeks	C57BL/6 male mice	OGTT	ZT9 on day 4	glucose tolerance ↓	Figueiro et al. 2017 [14]
10 h delay on day 1-4, normal phase on day 5-7	12 weeks	Sudanian grass male rats	OGTT	ZT2; day NR	glucose tolerance ↓	Grosbellet et al. 2015 [15]
8 h advance on day 1-3, normal phase on day 4-6 (6-day cycles)	8 weeks	C57BL/6 female/male mice	OGTT	NR	glucose tolerance ↓	Thaiss et al. 2014 [16]
10 h advance on day 1-4, normal phase on day 5-7	20 weeks	Long-Evans male rats	OGTT	ZT2 on day 7	glucose tolerance unchanged	Bartol-Munier et al. 2006 [17]
6 h advance on day 1-3, 12 h advance on day 4-6, 18 h advance on day 7-9, normal phase on day 10-12 (12-day cycles)	10 weeks	Sprague Dawley male rats	hyperinsulinemic-euglycemic clamp	ZT2 on day 10	insulin sensitivity unchanged	Gale et al. 2011 [18]

14. Figueiro MG, Radetsky L, Plitnick B, Rea MS. Glucose tolerance in mice exposed to light-dark stimulus patterns mirroring dayshift and rotating shift schedules. Sci Rep. 2017;7:40661. Epub 2017/01/13. doi: 10.1038/srep40661. PubMed PMID: 28079162; PubMed Central PMCID: PMCPmc5227691.

15. Grosbellet E, Zahn S, Arrive M, Dumont S, Gourmelen S, Pevet P, et al. Circadian desynchronization triggers premature cellular aging in a diurnal rodent. FASEB J. 2015;29(12):4794-803. Epub 2015/08/12. doi: 10.1096/fj.14-266817. PubMed PMID: 26260033.

16. Thaiss CA, Zeevi D, Levy M, Zilberman-Schapira G, Suez J, Tengeler AC, et al. Transkingdom control of microbiota diurnal oscillations promotes metabolic homeostasis. Cell. 2014;159(3):514-29. Epub 2014/11/25. doi: 10.1016/j.cell.2014.09.048. PubMed PMID: 25417104.

17. Bartol-Munier I, Gourmelen S, Pevet P, Challet E. Combined effects of high-fat feeding and circadian desynchronization. Int J Obes (Lond). 2006;30(1):60-7. Epub 2005/09/15. doi: 10.1038/sj.ijo.0803048. PubMed PMID: 16158090.

18. Gale JE, Cox HI, Qian J, Block GD, Colwell CS, Matveyenko AV. Disruption of circadian rhythms accelerates development of diabetes through pancreatic beta-cell loss and dysfunction. J Biol Rhythms. 2011;26(5):423-33. Epub 2011/09/17. doi: 10.1177/0748730411416341. PubMed PMID: 21921296; PubMed Central PMCID: PMCPmc3359760.
