# Supplementary material for: Circadian misalignment alters insulin sensitivity during the light phase and shifts glucose tolerance rhythms in female mice
Source: PLoS One. 2019 Dec 18;14(12):e0225813. doi: 10.1371/journal.pone.0225813 (PMC6919582; doi:10.1371/journal.pone.0225813)
Supplement: S2 Table — (PDF) [file pone.0225813.s003.pdf]

Gene	Forward primer (5'-3')	Reverse primer (5'-3')
G6pase	GCATTTGCCAGGAAGAGAAAG	AACTGAAGCCGGTTAGACATAG
PEPCK	TTTGTAGGAGCAGCCATGAG	CCGAAGTTGTAGCCGAAGAA
Glut2	CTTACAGTCACACCAGCATACA	AGACAGAGACCAGAGCATAGT
Pygl	GACATCTTCCTCCGGGAAATAG	CATGGAGGATGGGTTGATCTT
Gys2	GCTGTGGATGCGATGAATAAAC	CTGAGGAGCTGATGTCAAAGAG
Gck	CATCAGGAGGCCAGTGTAAAG	TCCCAGGTCTAAGGAGAGAAAG
Foxo1	GAGCCTCCTTCAAACAGAGTAG	GCAATGGTAAGAAATGGCAGAG
Pparα	AACCTGAGGAAGCCGTTCTGTGACAT	GACCAGCTGCCGAAGGTCCACCAT
Pparγ	CACAATGCCATCAGGTTTGG	GCTGGTCGATATCACTGGAGATC
PGC-1α	CTAGCCATGGATGGCCTATTT	GTCTCGACACGGAGAGTTAAAG
Clock	GAGTGTTCAGTCCCTTGATGAG	GCTGTGTCCCTATGACCATTT
Bmal1	CAACCCATACACAGAAGCAAAC	CATCTGCTGCCCTGAGAATTA
Cry1	CTCAGTCCTTATCTCCGCTTTG	CCACAGGAGTTGCCCATAAA
Cry2	AGGTGTGGTGTGCCATTT	GCAGCCCTGGTAAGAAGATAC
Per1	CCTGGAGGAATTGGAGCATATC	CCTGCCTGCTCCGAAATATAG
Per2	CAACAACCCACACACCAAAC	CTCGATCAGATCCTGAGGTAGA
Rev-erbα	GGGCACAAGCAACATTACCA	CACGTCCCCACACACCTTAC
RORα	CACGAGTGAGAGACAAGCATAA	CTTTGATGCCAGACCAGTATCT
β-actin	GAGGTATCCTGACCCTGAAGTA	CACACGCAGCTCATTGTAGA
