# Supplementary material for: Circadian misalignment alters insulin sensitivity during the light phase and shifts glucose tolerance rhythms in female mice
Source: PLoS One. 2019 Dec 18;14(12):e0225813. doi: 10.1371/journal.pone.0225813 (PMC6919582; doi:10.1371/journal.pone.0225813)
Supplement: S3 Table — All values are shown as mean±SD, n = 30 for each group. (PDF) [file pone.0225813.s004.pdf]

Variables	Control	Shift work
Body weight (g)	19.44±0.77	19.58±1.20
Body weight gain (%)	8.74±3.28	8.34±4.53
Liver weight/ body weight (%)	4.31±0.31	4.29±0.25
Gonadal WAT weight/ body weight (%)	0.90±0.25	0.89±0.30
Pancreas weight/ body weight (%)	1.74±0.28	1.68±0.35
