# Supplementary material for: Circadian misalignment alters insulin sensitivity during the light phase and shifts glucose tolerance rhythms in female mice
Source: PLoS One. 2019 Dec 18;14(12):e0225813. doi: 10.1371/journal.pone.0225813 (PMC6919582; doi:10.1371/journal.pone.0225813)
Supplement: S4 Table — Values are mean±SD. Mesor(A): midline estimating statistic of rhythm; Amplitude(B): half of the curve variation range; Phase(C): the timing of curve maximum (unit is hours). (PDF) [file pone.0225813.s005.pdf]

	Group	*P* value of rhythmicity	Mesor, A	Amplitude, B	Phase (hour), C
Day1	Control	<0.0001	0.69±0.04	0.57±0.05	17.24±0.34
	Shift work	<0.0001	0.72±0.05	0.66±0.07	14.64±0.38
Day2	Control	<0.0001	0.72±0.03	0.60±0.04	17.69±0.25
	Shift work	<0.0001	0.85±0.04	0.45±0.05	12.84±0.45
Day3	Control	<0.0001	0.78±0.03	0.60±0.04	16.75±0.28
	Shift work	<0.0001	0.81±0.04	0.50±0.06	11.48±0.47
Day4	Control	<0.0001	0.80±0.04	0.71±0.06	16.07±0.31
	Shift work	<0.0001	0.79±0.04	0.53±0.05	11.58±0.39
Day5	Control	<0.0001	0.89±0.05	0.79±0.07	15.59±0.31
	Shift work	<0.0001	0.84±0.04	0.67±0.06	13.08±0.34
Day6	Control	<0.0001	0.90±0.05	0.87±0.07	15.84±0.31
	Shift work	<0.0001	0.80±0.04	0.58±0.05	13.98±0.32
Day7	Control	<0.0001	0.85±0.05	0.82±0.07	15.43±0.32
	Shift work	<0.0001	0.90±0.03	0.56±0.05	14.92±0.32
