# Supplementary material for: Circadian misalignment alters insulin sensitivity during the light phase and shifts glucose tolerance rhythms in female mice
Source: PLoS One. 2019 Dec 18;14(12):e0225813. doi: 10.1371/journal.pone.0225813 (PMC6919582; doi:10.1371/journal.pone.0225813)
Supplement: S5 Table — All values are shown as mean±SD, n = 4 for each group. Light phase represented the period of ZT0–12; Dark phase represented the period of ZT12–24. (PDF) [file pone.0225813.s006.pdf]

	Control	Shift work
24-hour total movement distance (m)	505.23±132.13	499.61±101.58
Light-phase movement distance (m)	95.79±40.60	94.35±81.32
Dark-phase movement distance (m)	409.44±114.62	405.26±150.91
