# Supplementary material for: Circadian misalignment alters insulin sensitivity during the light phase and shifts glucose tolerance rhythms in female mice
Source: PLoS One. 2019 Dec 18;14(12):e0225813. doi: 10.1371/journal.pone.0225813 (PMC6919582; doi:10.1371/journal.pone.0225813)
Supplement: S6 Table — The comparisons of gene expression levels between two groups were performed with two-factor analysis of variance (time*treatment) and Bonferroni's post-hoc tests. *P<0.05. (PDF) [file pone.0225813.s007.pdf]

Gene	Time		Treatment		Time*treatment	
	F value	*P* value	*F* value	*P* value	*F* value	*P* value
G6pase	17.895	<0.001*	0.209	0.650	0.640	0.670
PEPCK	16.237	<0.001*	0.348	0.558	2.029	0.091
Glut2	33.814	<0.001*	1.329	0.255	1.640	0.168
Pygl	21.820	<0.001*	1.316	0.257	1.340	0.264
Gys2	33.824	<0.001*	0.353	0.555	2.389	0.052
Gck	13.738	<0.001*	0.414	0.523	1.968	0.101
Foxo1	18.336	<0.001*	0.073	0.788	2.706	0.031*
Pparα	52.329	<0.001*	0.422	0.519	3.207	0.014*
Pparγ	6.391	<0.001*	0.176	0.676	3.164	0.015*
PGC-1α	11.262	<0.001*	0.142	0.708	1.749	0.142
Clock	62.720	<0.001*	0.171	0.681	5.802	<0.001*
Bmal1	104.541	<0.001*	0.823	0.369	3.231	0.014*
Cry1	64.237	<0.001*	3.957	0.052	4.987	0.001*
Cry2	31.305	<0.001*	0.242	0.625	2.297	0.060
Per1	15.330	<0.001*	0.114	0.737	1.635	0.169
Per2	67.879	<0.001*	0.311	0.580	6.687	<0.001*
Rev-erbα	67.679	<0.001*	2.527	0.118	3.883	0.005*
RORα	26.691	<0.001*	0.397	0.532	3.858	0.005*
